# A novel pyroptosis-related indicator of immune infiltration features and prognosis in breast cancer

**DOI:** 10.3389/fonc.2022.961500

**Published:** 2022-09-07

**Authors:** Cheng Wang, Liyong Zhang, Lin Ren, Guozhi Zhang, Andi Wan, Siyi Xiong, Hao Tian, Zaihui Peng, Tingting Zhao, Pingping Gao, Na Sun, Yi Zhang, Xiaowei Qi

**Affiliations:** Department of Breast and Thyroid Surgery, Southwest Hospital, Army Medical University, Chongqing, China

**Keywords:** breast cancer, pyroptosis, prognosis, immunity, survival

## Abstract

Breast cancer is the most common malignancy in women, and there is evidence for the dual role of cell pyroptosis in tumor development. However, little is known about the relationship between cell pyroptosis and breast cancer and its prognostic value. We aimed to construct a prognostic model using cell-pyroptosis-related genes to provide innovative insights into the prognosis and treatment of breast cancer. We screened candidate genes for pyroptosis using public databases and identified 10 cell pyroptosis signature genes with the random forest method. Finally, a nomogram for predicting 1-, 3-, and 5-year survival probabilities was constructed. The differences in immune cell distributions between survival periods were similar across the breast cancer datasets. The 10 identified key pyroptosis factors showed a significant correlation with Her2, tumor–node–metastasis (TNM) stage, and survival of breast cancer. The risk scores correlated positively with the infiltration features of naive B cells, CD8+ T cells, atpdelnd mast cells, while they correlated negatively with those of M0 macrophages and dendritic cells. In conclusion, our findings confirm that cell pyroptosis is closely associated with breast cancer. Importantly, the prognostic complex values generated from the 10 cell-pyroptosis-related genes based on various clinical features may provide an important basis for future studies on the prognosis of breast cancer.

## Introduction

According to the latest data on the global cancer burden, in 2020, breast cancer had the highest morbidity worldwide and was the leading cause of cancer-related deaths, with approximately 2.26 million newly diagnosed cases and 685,000 reported deaths (1). Among women, breast cancer accounts for 24.5% of all cases and 15.5% of cancer-related deaths; it ranks first among cancers in terms of incidence and cancer-related mortality in most countries (2). Owing to the continuous progress in medical treatment, the survival rate of patients with breast cancer has improved greatly, and the current 1-, 3-, and 5-year survival rates are 0.92, 0.75, and 0.73, respectively. Although the survival rate associated with breast cancer is better than that for most other tumors, the 10-year survival rate remains low (0.61), and some patients experience distant recurrence at different times, with an average survival time of 2 years after recurrence (3). Given this situation, a reliable prognostic model may help improve the survival rate of patients.

Cell pyroptosis, also known as cellular inflammatory necrosis, is an inflammatory form of programmed cell death (PCD), but it differs considerably from other types of PCD (4). It was first identified in infected macrophages in 1992 (5), and the term was coined by Cookson et al. (6). PCD induced under various stimuli has been studied extensively in many disease models, and cell pyroptosis was found to be mediated by gasdermin (GSDM) (7). The GSDM family includes gasdermin A (GSDMA), gasdermin B (GSDMB), gasdermin C (GSDMC), gasdermin D (GSDMD), gasdermin E (GSDME), and PJVK (8). Except for PJVK, the other members of the GSDM family have both C- and N-terminal structural domains. GSDM is cleaved, and the GSDM-N structural domain is released (9). The released N-terminal domain perforates the cell membrane and leads to characteristic morphological changes associated with pyroptosis, including cytoplasmic swelling, membrane rupture, and release of inflammatory factors into the extracellular environment, thus directly amplifying the systemic immune responses (9). The main manifestation of pyroptosis in cells is swelling that results in cell membrane rupture, which leads to the efflux of contents and activation of an intense inflammatory response (10).

The role of cell pyroptosis in cancers is gradually becoming evident. In esophageal squamous carcinoma, head and neck squamous carcinoma, and hepatocellular carcinoma, pyroptosis can be induced by different drugs to achieve tumor suppression (11–13). On the other hand, pyroptosis inhibition protects microglia and neurons, rescues dopaminergic neurons, inhibits neuroinflammation, and alleviates neurodegeneration (14). Therefore, pyroptosis is a double-edged sword that plays a key role in antitumor immunity in certain tumors and may provide an effective treatment strategy for cancer; however, its induction in normal tissues and immune cells can lead to severe damage (15). Thus, in the case of tumors, on the one hand, pyroptosis causes inflammation that contributes to the generation and maintenance of an inflammatory microenvironment around cancer cells, thus promoting tumor progression; on the other hand, acute activation of pyroptosis leads to the infiltration of multiple immune cell types that inhibit tumor progression (16). In non-small cell lung cancer, GSDME-mediated pyroptosis can make immune factors MIP-1α, MIP-1β, MIP-2, and IP-10 increase, thereby recruiting T cells to achieve anti-tumor effect (17). Some chemotherapeutic drugs can induce the expression of GSDMC and activate caspase-8 and induce the pyroptosis of breast cancer cells (18). Pyroptosis also has different anti-tumor mechanisms in the study of hepatocellular carcinoma, gastric cancer, and ovarian cancer (19). To examine the prognostic impact of pyroptosis, a pyroptosis score was constructed for melanomas. The results indicated that cell pyroptosis is an independent prognostic factor that can improve survival by strong immune clearance in tumors through immune cells, including T cells, B cells, and natural killer (NK) cells (20). Furthermore, a recent study found that N-oxide induced pyroptosis in tumor cells by activating the endoplasmic reticulum stress kinase PERK and, thus, enhanced CD8+ T-cell-mediated antitumor immunity in triple-negative breast cancer (TNBC) in the *in vivo* setting (21). All these findings reveal the close relationship between pyroptosis and breast cancer. Specifically, cellular pyroptosis plays an important role in tumorigenesis and antitumor processes, but its specific function in breast cancer is unclear.

This systematic study was conducted to determine the expression of genes associated with cell pyroptosis in normal breast tissues and breast cancer tissues in order to evaluate their prognostic value and further investigate the correlation between cell pyroptosis and the tumor immune microenvironment. We aimed to comprehensively assess the relationship between cell pyroptosis and the prognosis of breast cancer patients and also constructed a nomogram based on breast cancer pyroptosis-related genes. The efficacy of this nomogram was evaluated for prognostic prediction, molecular characterization, clinical significance, and assessment of the regulatory function of the immune microenvironment.

## Material and methods

### Data collection

The Cancer Genome Atlas Breast Cancer (TCGA-BRCA) expression profile, variants, clinical information, and follow-up data were downloaded from XENA, and samples with complete phenotypic and survival data were retained for further analyses. The GSE96058 expression data and corresponding sample information were downloaded from the GEO database. The METABRIC expression data and corresponding sample information were downloaded from the ciBoportal database. The data were preprocessed as follows: first, probes corresponding to genes according to the annotation file were included, while unannotated probes were removed. If there were multiple probes that corresponded to the same gene, the probe with the maximum value was considered to represent the level of expression of that gene. All genes with low expression were filtered out at a cutoff of >1 in at least 10% of the samples.

### Differential gene analysis

Differential analysis of data normalized to fragments per kilobase of transcript per million (FPKM) in TCGA-BRCA was performed using the limma package in R. For screening, the criteria were set as |FC| > 1.5 and *p* < 0.05.

### Cox regression and survival analyses

For Cox univariate analysis, regression modeling of individual genes or clinical features [age, tumor–node–metastasis (TNM) stage, LumA, LumB, TNBC, and Her2 status] was performed using the coxph function in the survival package. Prognosis-related genes or clinical features were screened at a cutoff *p*-value of <0.05. After the corresponding modeling parameters were extracted, forest plots were drawn using the forestplot package.

For survival analysis, related genes were screened and grouped according to their median expression level. Overall survival (OS) and grouping information were fitted using the survfit function in the surv package and finally analyzed and visualized using the ggsurvplot function in the survminer package.

### Screening of pyroptosis factors and gene set variation analysis by the random forest method

Potential pyroptosis-related factors were screened using the random forest (RF) algorithm. A three-time and 10-fold crossover model was constructed using the carat package. The optimized parameters were input into the RF package, and finally, genes with the top 10 MeanDecreaseGini scores were selected. The pROC package was used for prognosis prediction of the tumor samples, and receiver operating characteristic (ROC) curves were drawn. Finally, the genes with the top 10 MeanDecreaseGini scores were selected as the key pyroptosis factors.

The pyroptosis signature gene expression values were determined from normalized expression data in TCGA-BRCA, and gene set variation analysis (GSVA) was performed on the normalized expression data using the GSVA package to obtain the complex values.

### Prediction of survival using the prognostic complex

Regression modeling of the complex values was performed using the coxph function in the survival package, and the samples were classified into high- and low-risk groups according to the median of the complex values. Finally, OS was analyzed.

### Validation of the prognostic complex and nomogram construction

In order to verify the independent prognostic efficacy of the high and low complex values, univariate Cox analysis was first performed on the TCGA-BRCA dataset in combination with other clinicopathological characteristics, including age, TNM stage, LumA, LumB, TNBC, and Her2 status. Next, overall prognosis based on the above factors was analyzed by multi-factor Cox regression analysis to verify the independent prognostic efficacy of the risk score. The Cox proportional hazards regression model was constructed using the cph function in the R package rms. Finally, the survival package was used to calculate survival probability, and the nomogram function was used to construct the nomogram and plot the calibration curves to evaluate its accuracy and predict its utility.

### Immune infiltration analysis

The immune infiltration score files for TCGA were downloaded from the TIMER2 (http://timer.comp-genomics.org/) database, and TCGA-BRCA sample data were screened. The corresponding CIBERSORT score data were used for comparison of immune cell differences among samples with different survival values, using the t_test function in the rstatix package in R. Difference analysis and correlation analysis between the complex value and immune cell proportions were conducted by the Pearson method. Immune infiltration scoring was performed using TIMER2 along with normalized expression data from GSE96058 and METABRIC. Finally, immune cell differential analysis and mapping were performed.

### Enrichment analysis and correlation analysis of key genes associated with the clinical features

The enrichment of Gene Ontology (GO) terms and Kyoto Encyclopedia of Genes and Genomes (KEGG) pathways was analyzed using the clusterProfiler package. The enriched GO terms were further analyzed by GSEA. For GO terms that were enriched in both steps, similarity calculation and hclust clustering were performed using the GOSemSim package.

For screening tumor samples for TCGA molecular typing and stage, mosaic correlation analysis was performed on survival-related clinical information, and the expression levels of pyroptosis-related key genes were analyzed with the vcd package.

### Mutation analysis and molecular docking

The Mutect2 mutation files of TCGA-BRCA were downloaded using the R package, TCGAbiolinks, and this was followed by visualization of mutation types using the R package maftools. The corresponding compound structures were downloaded from the DrugBank database (https://go.drugbank.com/) and screened according to Lipinski’s rules (hydrogen bond acceptor ≤ 10, hydrogen bond donor ≤ 5, rotatable bond ≤ 10, the logarithmic value of lipid–water partition coefficient ≤ 5, molecular weight 180–480, and polar surface area ≤ 140). Finally, 5,464 small-molecule compounds were obtained. The 3D structural information of the proteins encoded by the characteristic pyroptosis-related genes in breast cancer was queried in PDB (https://www1.rcsb.org/). Relevant structural information was obtained for PAK7, and its corresponding PDB file, 2F57, was downloaded. The approximate docking box range was identified according to the ligand information therein, and other relevant parameters of AutoDock Vina were set. Docking with small-molecule compounds was performed with AutoDock Vina, and demonstration and analysis of interaction forces were performed using Pymol and Ligplus.

### Drug sensitivity analysis

We analyzed the drug resistance of genes based on the GDSC drug database. The mRNA expression data and drug sensitivity data were merged. Pearson correlation analysis was performed to determine the correlation between mRNA expression of the screened pyroptosis-related genes and the IC50 values of drugs. The *p*-value was adjusted by false discovery rate (FDR).

### Codes

The codes used to analyze the data can be found here: https://github.com/wzhlc1206/ A-Novel-Pyroptosis-Related-Signature-Indicates-Immune-infiltration-Features-and-Predicts-Prognosis-.git

## Results

### Identification of pyroptosis-related genes

**Figure 1:**
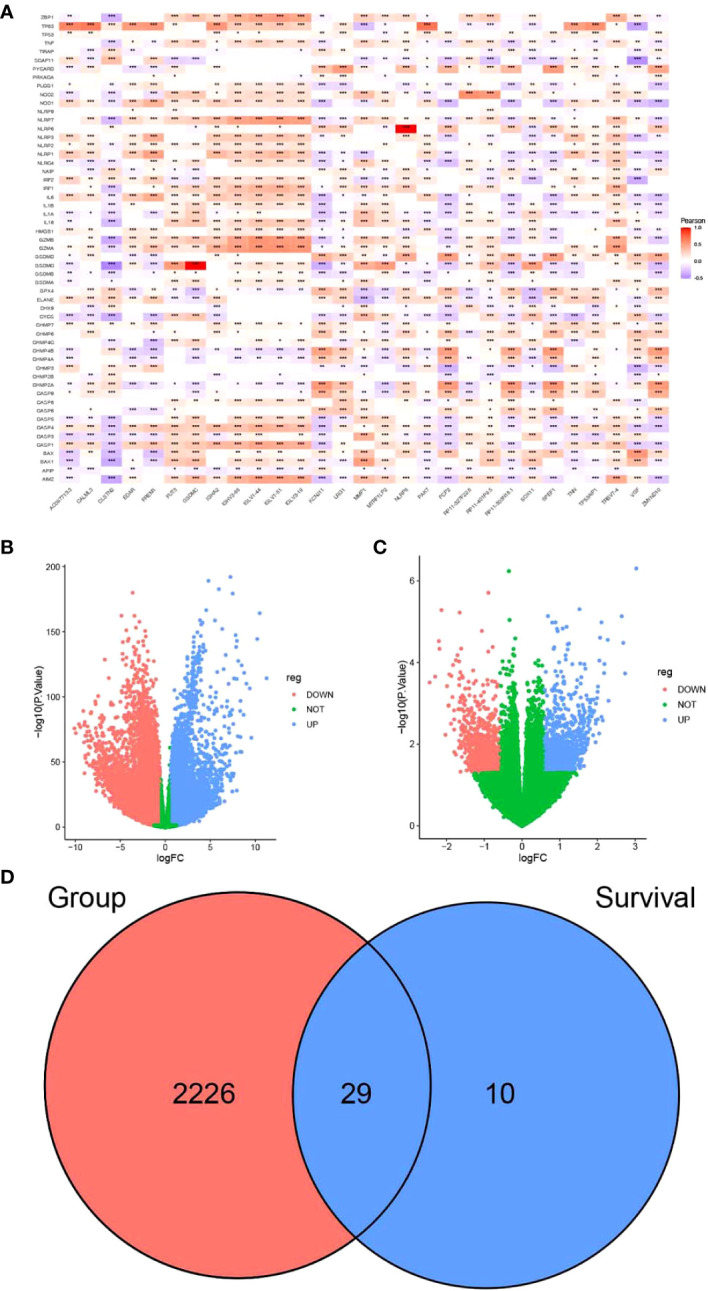
Screening of candidate genes for pyrosis. **(A)** Heatmap of the correlation between partial differences in the TCGA-BRCA dataset and pyroptosis genes. **(B)** Volcano plot depicting the results of differential analysis between tumor and normal datasets and **(C)** survival time. **(D)** Venn diagram of intersecting pyroptosis genes. *P < 0.05, **P < 0.01, ***P < 0.001.

First, we combined the pyroptosis-related genes from the PMID:338828074, MsigDB, and GeneCards databases (**Supplementary Table S1**) and obtained a pyroptosis gene set containing 57 genes (**Supplementary Table S2**). Next, the correlation of these genes with the normalized expression data in the TCGA-BRCA dataset was analyzed. Genes with Pearson correlation coefficients >0.6 and p-values <1e−10 were selected as candidate pyroptosis-related genes. Finally, 4,939 genes and 4,996 pyroptosis-related genes were obtained. The heatmap of partial genes is shown in **Figure 1A**, and the RNA expression profile for breast cancer is shown in **Supplementary Table S3**.

FPKM expression data from TCGA-BRCA were processed using limma and filtered to exclude genes with low expression levels. These values were normalized for principal component analysis (PCA). Short-term survival was set as <5 years, while long-term survival was set as more than 5 years. By comparing the expression of differentially expressed genes, we found that there were significant differences between normal and tumor samples (**Supplementary Figure S1A**). In contrast, differences in short- and long-term survival were less significant (**Supplementary Figure S1B**).

Differential analysis of TCGA-BRCA data was performed using the limma package according to the presence or absence of disease. Differential genes were screened between breast cancer and normal tissue samples (**Supplementary Table S4**). The genes that were associated with survival are listed in **Supplementary Table S5**. The screening criteria were as follows: |FC| > 1.5 and *p* < 0.05. A total of 10,913 genes were upregulated, while 10,577 were downregulated in breast cancer samples as compared to the normal samples (**Figure 1B**). A total of 2,255 intersecting genes related to pyroptosis were obtained (**Figure 1D**). The breast cancer samples were grouped according to survival time for differential analysis: 994 genes were upregulated, while 906 were downregulated in patients with long-term survival (**Figure 1C**). The last two sets of intersecting genes were overlapped, and 29 candidate genes for pyroptosis were identified (**Figure 1D**).

### Screening of core genes associated with pyroptosis

**Figure 2:**
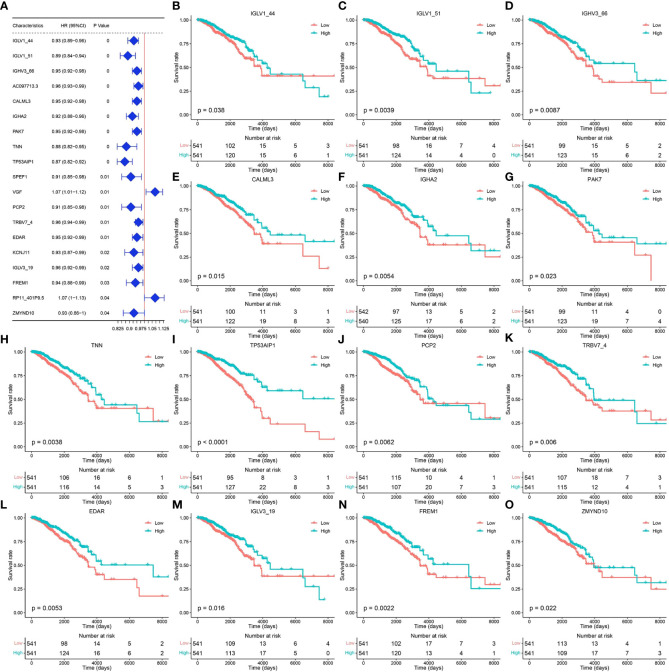
Genes related to the prognosis of breast cancer. **(A)** Results of univariate Cox analysis of the TCGA-BRCA dataset. **(B–O)** Prognosis-related pyroptosis gene expression and breast cancer survival curve.

Tumor samples in the TCGA-BRCA dataset were screened, and univariate Cox regression analysis was performed for 29 pyroptosis-related genes using the survival package. Based on the median gene expression values, the genes were classified into high- or low-expression groups. The results showed that a total of 19 genes were associated with breast cancer prognosis (**Figure 2A**, *p* < 0.05). Survival analysis of these prognosis-related pyroptosis genes based on the median threshold showed that 14 potential pyroptosis-related genes were significantly associated with survival in breast cancer: IGLV1-44, IGLV1-51, IGLV1-66, CALML3, IGHA2, PAK7, TNN, TP53AIP1, PCP2, TRBV7-4, EDAR, IGLV3-19, FREM1, and ZMYND10 (**Figures 2B–O**). High expression of these genes for up to 6,000 days was associated with better survival, and these results were highly significant for TP53AIP1.

**Figure 3:**
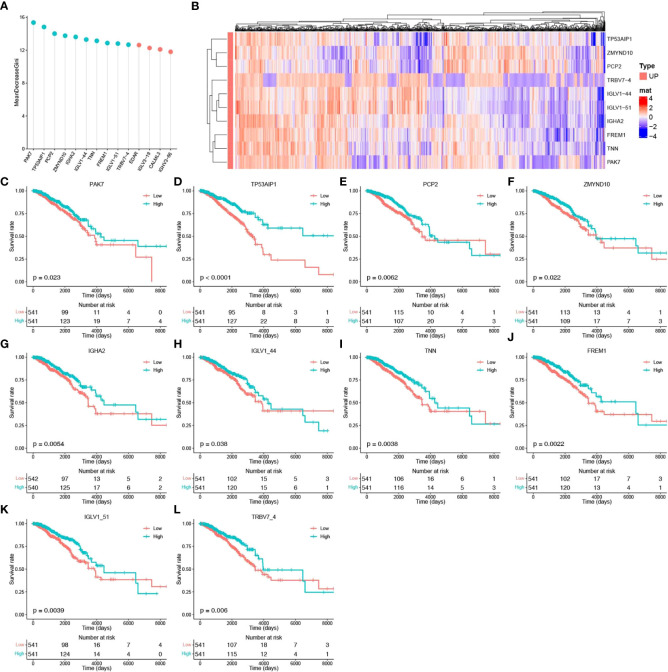
Identification of core pyroptosis-related genes and relationship between the expression of these genes and survival. **(A)** MeanDecreaseGini score after RF modeling of potential scores (light blue indicates the top 10 pyroptosis factor scores). **(B)** Heat map of the association between the level of expression of the top 10 pyroptosis factors and breast cancer survival. **(C–L)** Survival analysis of the pyroptosis factors.

To identify the core genes associated with pyroptosis, we further screened these 14 potential pyroptosis genes. A three-time 10-fold crossover model was constructed using the carat package, and finally, the normalized dataset was analyzed using the RF method with optimized parameters. Gini scoring was performed, and finally, the top 10 genes according to their MeanDecreaseGini scores were selected as the core genes associated with pyroptosis. These genes included PAK7, TP53AIP1, PCP2, ZMYND10, IGHA2, IGLV1-44, TNN, FREM1, IGLV1-51, and TRBV7-4. The potential pyroptosis-related genes according to their MeanDecreaseGini scores are shown in **Figure 3A**. The heat map for their expression is shown in **Figure 3B**, and **Figures 3C–L** show the expression of these genes, along with survival, in breast cancer patients.

### Prediction of OS based on prognostic complex value and validation of independent prognostic factors

The pyroptosis-related genes identified after screening were used as the defined set for GSVA. GSVA was performed for breast cancer samples using the GSVA package, and the complex prognostic value was obtained. The samples were then divided into high- or low-risk groups according to the median complex prognostic values. The correlation between survival and pyroptosis-related gene expression was determined. Significant differences in survival and LumB, LumA, and Her2 subtypes were found between the high- and low-risk groups. Association analysis of survival and expression was performed for the overall sample and five subtypes according to LumB, Basal, LumA, Normal, and Her2 status (**Supplementary Figures 2A–F**), based on the complex prognostic values calculated using GSVA for breast cancer samples.

**Figure 4:**
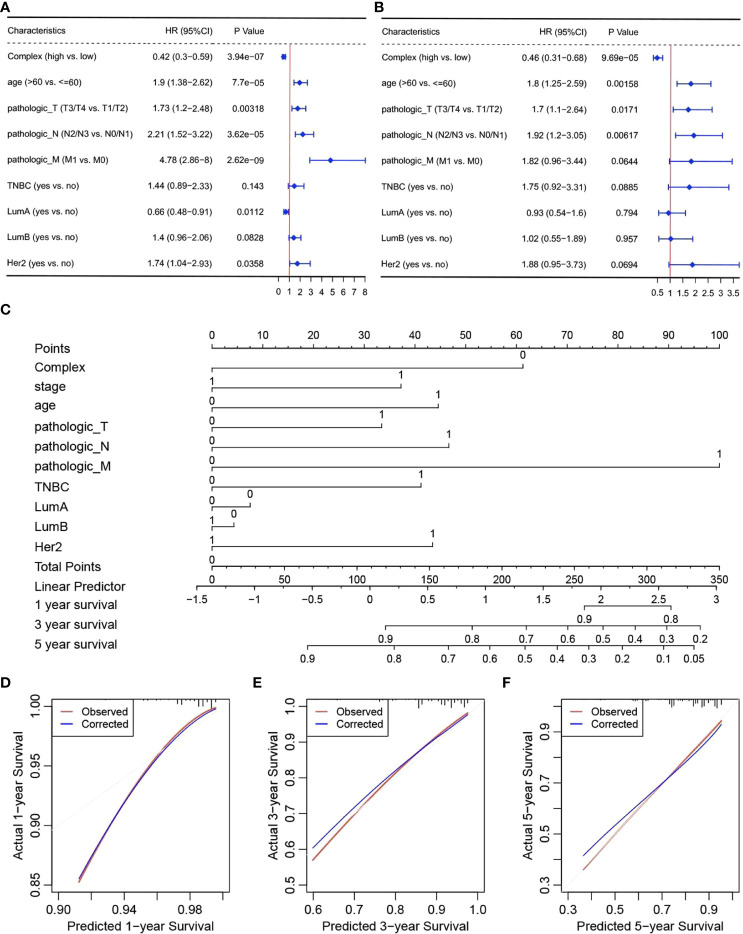
Independent prognostic efficacy of complex prognostic values. Results of **(A)** univariate and **(B)** multivariate Cox regression analyses, **(C)** nomograms, and calibration curves for survival at **(D)** one **(E)** 3 and **(F)** 5 years.

To verify the independent prognostic efficacy of the high and low complex prognostic values, Cox univariate analysis was first performed on the TCGA-BRCA dataset in combination with other prognostic factors, including stage, age, Her2, LumA, LumB, TNBC status, and TNM stage (**Figure 4A**). Overall prognosis based on the above six factors (including the complex prognostic values) was then analyzed by multifactor Cox regression (**Figure 4B**). The results confirmed that the complex values were independent prognostic factors and could be used to develop prognostic column line plots that could predict the probability of survival at 1, 3, and 5 years. Nomograms of 1-, 3-, and 5-year survival probabilities were verified by the calibration graph method, and the standard curve of the calibration graph was in good agreement with the calibration prediction curve (**Figures 4C–F**). The predicted and observed values for 1-, 3-, and 5-year survival were in good agreement.

### Correlation analysis of complex prognostic values and immune-infiltrating cells

**Figure 5:**
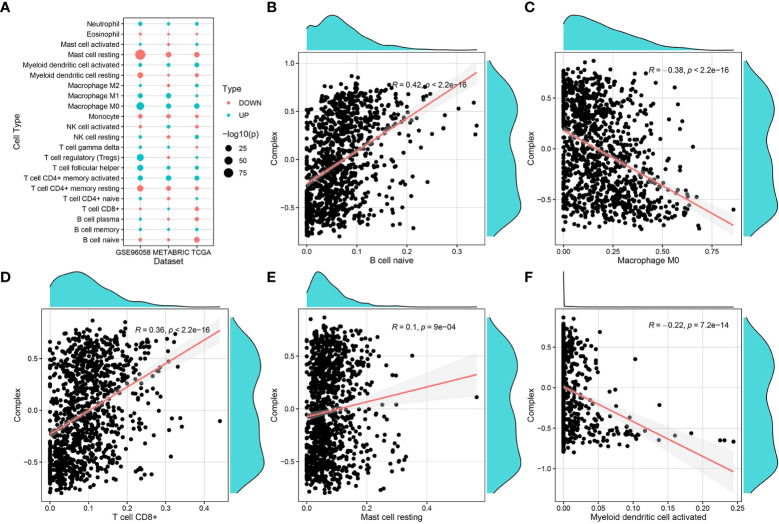
Correlation of complex prognostic values and immune infiltrating cells. **(A)** Differences in the distribution and proportion of immune cells in the TCGA-BRCA, GSE96058, and METABRIC datasets. **(B–F)** Correlation analysis of complex values with the proportion of different immune cell types.

The immune infiltration score files for TCGA were downloaded from the TIMER2 database, and data related to the breast cancer samples in TCGA-BRCA were selected and grouped according to survival (with 5 years set as the threshold). The CIBERSORT score data were used for immune cell differential analysis and mapping (**Figure 5A**). Immune infiltration scoring was performed using TIMER2 for standardized expression data from GSE96085 and METABRIC, followed by immune cell differential analysis and mapping. The results of immune infiltration analysis showed that the complex values were positively correlated with infiltration of naive B cells, CD8+ T cells, and mast cells, while they were negatively correlated with M0 macrophages and dendritic cells (**Figures 5B–F**).

### Enrichment analyses of key functional genes and pathways

**Figure 6:**
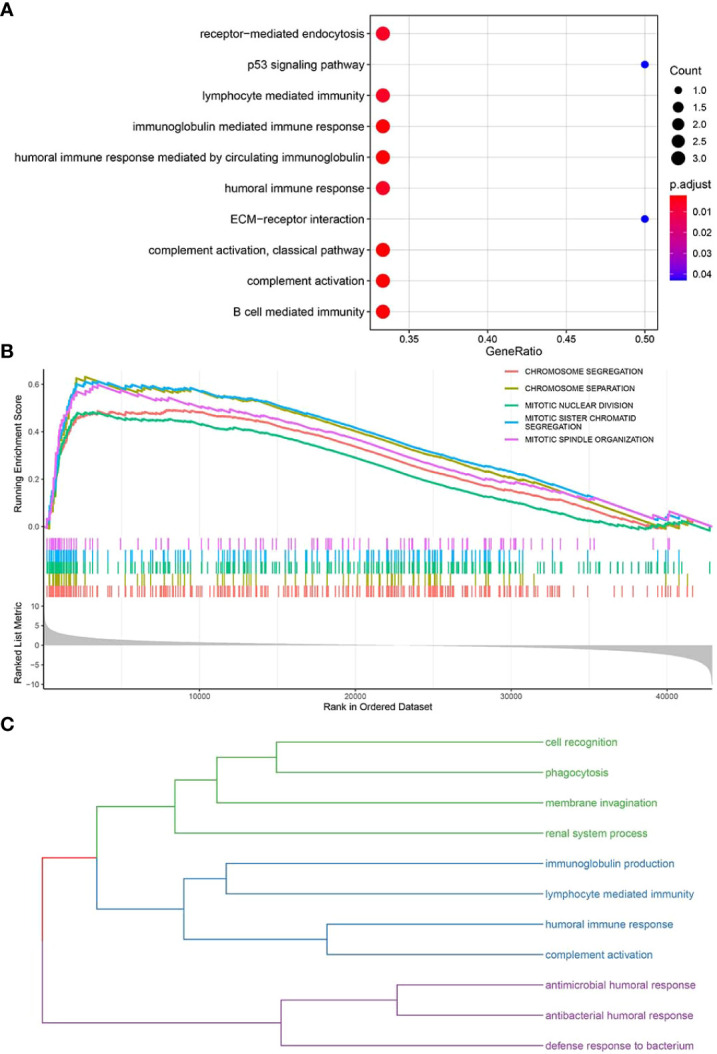
Enrichment analyses of key functional genes and pathways. **(A)** Bubble plot for enriched GO-BP and KEGG pathways related to key pyroptosis factors. **(B)** GSEA for enriched pathways in the TCGA-BRCA samples. **(C)** The enriched pathways in panels **(A, B)** are classified into three major categories of biological functions based on similarity.

The clusterProfiler package was used to perform GO-BP (**Supplementary Table S6**) and KEGG pathway enrichment analyses (**Supplementary Table S7**) (**Figure 6A**) for the top 10 pyroptosis factors identified with the RF method and enriched pathways from GSEA (**Supplementary Table S8**) (**Figure 6B**). For BPs enriched in the first two steps, similarity calculation and hclust clustering were performed using the GOSemSim package. Immune responses, defense responses, and cell recognition were enriched (**Figure 6C**); this indicates that these genes were involved in regulating similar BPs.

### Mutation analysis of key genes and their correlation with clinical features

**Figure 7:**
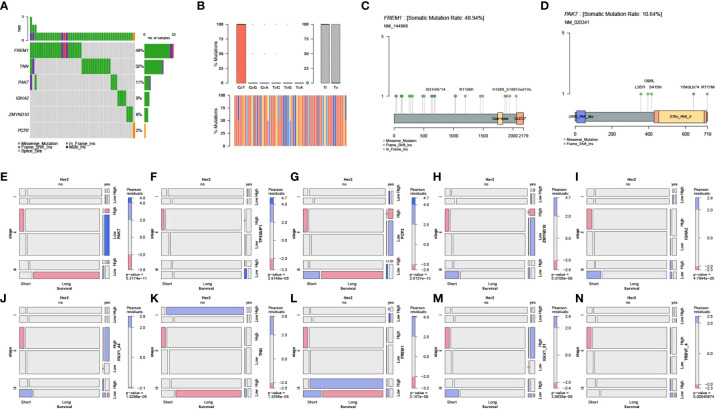
Mutation analysis of key genes and their correlation with clinical features. **(A)** Waterfall plot for point mutation analysis of key pyroptosis factors in samples from the TCGA-BRCA dataset. **(B)** Statistical plot of point mutation types. **(C, D)** Point mutation distribution for FREM1 and PAK7 and **(E–N)** the relationship between key pyroptosis factors and TCGA molecular typing, Her2 status, and stage of breast cancer.

The TCGA-BRCA point mutation maf files of Mutect analysis were downloaded from TCGA. The mutation data of tumor samples and pyroptosis core genes were screened, and this was followed by visualization of mutation types using the R package mafTools. Mutation analysis showed that FREM1, TNN, and PAK7 were the top 3 key pyroptosis genes with the highest mutation frequency (**Figures 7A–D**).

Screening of clinical information related to TCGA molecular typing and mosaic correlation analysis of Her2 status, stage, survival, and levels of expression of the 10 key genes of pyroptosis in breast cancer samples using the vcd package showed that there was a significant correlation between clinical features and expression of the 10 pyroptosis genes (**Figures 7E–N**).

### Screening of potential therapeutic compounds based on molecular docking experiments

**Figure 8:**
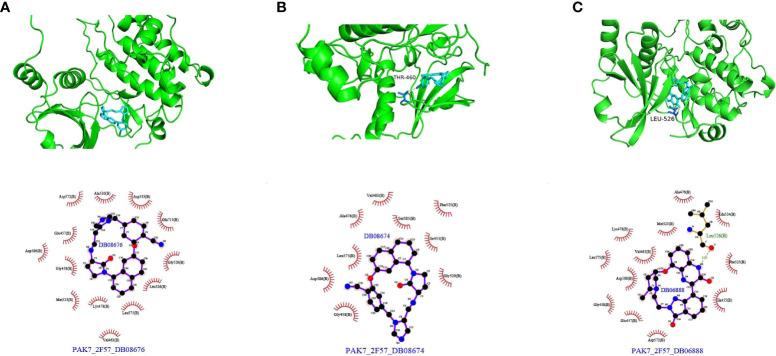
Docking conformation and interaction force analysis for PAK7 and three small-molecule compounds. **(A)** DB08676, **(B)** DB08674, and **(C)** DB06888. Top panel: Pymol shows the docking conformation and hydrogen bonding, wherein cyan is the small molecule, the yellow dashed line is the hydrogen bond, and blue is the amino acid residue forming the hydrogen bond with the small molecule. Bottom panel: Ligplus force analysis, wherein the small molecule is in the middle, surrounded by related protein amino acid residues; the green dashed line is the hydrogen bond; and the amino acid in green is the amino acid residue forming a hydrogen bond.

Protein structure information was downloaded from the PDB database. AutoDock Vina was used for molecular docking experiments. Compounds were filtered based on their binding energies, and finally, Pymol was used for drawing protein–compound binding maps. The corresponding compound structures were downloaded from the DrugBank database (https://go.drugbank.com/) and filtered according to Lipinski’s rule. Finally, 5,464 small-molecule compounds were obtained. 3D structural information about the proteins encoded by characteristic pyroptosis-related genes in breast cancer was obtained from the PDB database (https://www1.rcsb.org/), and only relevant structural information for PAK7 was obtained (the other genes did not have corresponding structural files containing ligands based on which the docking box range could be inferred). The corresponding PDB file, 2F57, was downloaded. The approximate docking box range was calculated based on the ligand information therein, and other relevant parameters were set for AutoDock Vina. AutoDock Vina was used for docking small-molecule compounds, and Pymol and Ligplus were used for demonstration and analysis of the interaction forces. The top 3 small-molecule compounds with the strongest binding abilities were DB08676, DB08674, and DB06888 (**Table 1**). Docking conformation and interaction force analysis of pak7 and these three small-molecule compounds are shown in **Figure 8**. The complete docking scores are provided in **Supplementary Table S9**.

**Table 1:** Top 10 compounds with the best docking scores with PAK7.

DrugBank ID	Hydrogen	Hydrogen	Rotatable	LogP	Molecular	TPSA	Affinity
	Acceptors	Donors	Bonds		Weight		(kcal/mol)
DB08676	5	1	0	2.5	453.5	83.2	-11.1
DB08674	5	1	0	2.8	435.5	83.2	-10.9
DB06888	6	2	0	2.2	403.4	86.3	-10.7
DB15054	5	2	3	2.5	424.5	86.2	-10.7
DB12012	8	1	4	3.3	455.4	80.2	-10.5
DB12200	2	2	2	2.9	369.4	66.9	-10.4
DB12523	5	1	5	3.2	432.5	65.8	-10.2
DB06469	4	3	1	2.2	439.5	88.6	-10.1
DB08683	3	1	0	3.8	393.4	65.3	-10.1
DB12611	5	4	4	1.5	419.5	130	-10.1

### Drug sensitivity analysis

**Figure 9:**
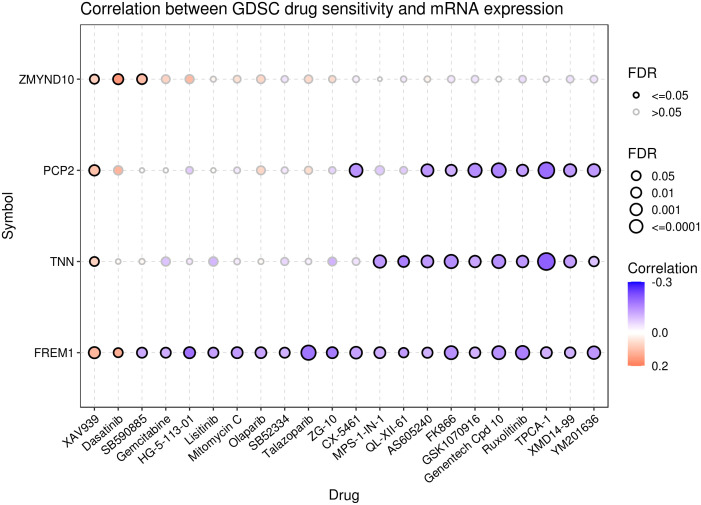
Drug sensitivity analysis of the pyroptosis genes. Data from the GSCA database represent the correlation between the expression of proptosis genes and drug sensitivity. A negative correlation indicates that patients with cancer with overexpression of the proptosis genes are sensitive to the drug, and vice versa.

To explore the relationship between pyroptosis genes and drug sensitivity, we analyzed the drug sensitivity of genes based on the GDSC drug database. The results showed that *ZMYND10*, *PCP2*, *TNN*, and *FREM1* were associated with drug response. Patients with cancer with high expression of the PCP2 and TNN genes were probably sensitive to AS605204 and FK886 and resistant to XAV939. Furthermore, cancers with high expression of the *FREM1* gene were sensitive to XAV939 and dasatinib and resistant to SB590885 and gemcitabine, and cancers with high expression of ZMYND10 were resistant to XAV939, gemcitabine, and SB590885 (**Figure 9**).

## Discussion

According to the latest statistics, breast cancer ranks first among tumors in women in terms of incidence (22), and the number of cases is expected to reach 4.4 million in 2070 (23). Increasing evidence shows that cell pyroptosis is closely associated with the development of lung, gastric, cervical, and breast cancers (24). Some scholars have used long-chain non-coding RNAs or lncRNAs associated with pyroptosis to predict the prognosis and tumor immune microenvironment of breast cancer patients (25); however, the role of pyroptosis-related genes in breast cancer remains unclear. Therefore, there is a need to examine the prognostic profiles of pyroptosis-related genes by combining independent prognostic factors associated with breast cancer. Herein, we describe the expression, mutation, immune infiltration, and clinical significance of pyroptosis-related genes in breast cancer.

In our study, most pyroptosis-related genes were differentially expressed in breast cancer, and this indicated their potential roles in tumorigenesis (26). We constructed a prognostic gene signature based on 10 cell pyroptosis genes (PAK7, TP53AIP1, PCP2, ZMYND10, IGHA2, IGLV1-44, TNN, FREM1, IGLV1-51, and TRBV7-4) and generated a nomogram for predicting the probability of survival at 1 and 3 years. Survival analysis showed significant differences in OS between high- and low-risk groups classified according to the complex prognostic values and between groups with high and low complex prognostic values for three breast cancer subtypes based on LumB, LumA, and Her2 status. The results of univariate and multifactorial Cox analyses revealed the independent prognostic effects of the complex prognostic values. Furthermore, the differences in immune cell distributions according to survival were similar across the different breast cancer datasets.

In the present study, the 10 key pyroptosis-related factors identified were significantly correlated with TCGA molecular typing based on the Her2 status and stage of breast cancer samples. Some scholars have pointed out that GSDMB overexpression/amplification occurs in 60% of HER2-positive breast cancers (27). In addition, higher expression of DRD2 was positively correlated with longer survival times, especially in HER2-positive patients, and DRD2 also triggered GSDME-mediated pyroptosis (28). This implies that HER2 may be more closely related to pyroptosis. Mutation analysis showed that FREM1, TNN, and PAK7 were the top 3 key pyroptosis genes with the highest mutation frequencies. Only structural data for PAK7 were available in the PDB database, and the results of molecular docking revealed that PAK7 had the strongest binding ability for the three small-molecule compounds examined, namely, DB08676, DB08674, and DB06888. Some studies have shown that small molecular drugs that can regulate immune cells will greatly improve the efficacy of cancer immunotherapy (29). Small molecular compounds can change the molecular pathway to deal with immune tolerance and overcome tumor-related immunosuppression, so as to produce effective anti-tumor response and improve the efficacy of cancer immunotherapy (30). Small molecular drugs are compatible with systemic administration and are suitable for extracellular and intracellular targets (31). In future research, we will pay more attention on how to improve the efficacy of breast cancer by combining immunotherapy with DB08676, DB08674, and DB06888 small molecular compounds. PAK7, an oncogene, is significantly upregulated in both gastric and esophageal squamous carcinoma (32, 33). Its expression is significantly enhanced in breast cancer tissues and correlates positively with its pathological differentiation and TNM stage. PAK7 is involved in breast cancer progression through the activation of the Wnt/β-linked protein signaling pathway; this indicates the potential applicability of PAK7 as a therapeutic target for the treatment of breast cancer (34). In fact, one study has reported that inhibition of osteosarcoma can be achieved by targeting PAK7 (35). Studies on non-small cell lung cancer suggest that the PAK7 mutant phenotype is also associated with the tumor immune microenvironment (36). Similarly, in our study, it was confirmed that PAK7 was correlated significantly with the TCGA molecular type, Her2 stage, TNM stage, and the immune microenvironment in breast cancer. Notably, TP53AIP1, ZMYND10, and FREM1 are associated with the apoptotic pathway (37). Moreover, apoptosis and pyroptosis are tightly linked to each other through multiple mechanisms (38). ZMYND10 suppresses breast cancer oncogenicity by inhibiting the miR145-5p/NEDD9 signaling pathway (39). As an oncogene, ZMYND10 promotes apoptosis in tumor cells by regulating the activity of sMEK1 (40) and inhibiting angiogenesis. In addition, ZMYND10 can upregulate BAX expression, which facilitates the pro-apoptotic pathway, thereby promoting paclitaxel-induced apoptosis in ovarian cancer cells. Reduced FREM1 expression is usually associated with hormone-receptor-negative and TNBC status, and correlates significantly with poor OS and recurrence-free survival. Furthermore, FREM1 expression correlates positively with the level of immune infiltration of CD4+, CD8+ T cells, and CD86+ M1 macrophages, while it correlates negatively with CD68+ pan− and CD163+ M2 macrophages. These findings suggest that FREM1 is a potential biomarker for assessing immune infiltration status and BC prognosis (41, 42). Interestingly, TILRR enhances IL-1-induced anti-apoptotic responses (43) and is a splice variant of the FRAS1 family (44). It has also been shown that high IGHA2 mRNA expression is associated with a more favorable prognosis (45); this is consistent with our findings. IGHA2 is significantly associated with the OS of patients with esophageal squamous cell carcinoma, as evaluated using a prognostic risk score model of immune-related genes (46). The close relationship between apoptosis and cell pyroptosis may explain the dual roles of TP53AIP1, ZMYND10, and FREM1. IGLV1-44 and IGLV1-51 are immune-related genes (47–49) that can influence immune infiltration. Our results suggest that several pathways associated with the pyroptosis-related genes were enriched. Thus far, the relationship of TNN, TRBV7-4, and PCP2 with cancer has not been reported, and our results suggest that these genes, as core pyroptosis genes, have important associations with breast cancer development and the immune microenvironment.

Breast cancer is often considered a cold tumor with reduced immune cell infiltration, suppressed immune microenvironment, and low mutation frequency (50). Importantly, immunotherapy may produce lasting therapeutic effects. Therefore, many scholars have studied and explored immunotherapy strategies for breast cancer. For example, atezozumab is a monoclonal antibody against PD-L1. In a phase III study, atezozumab combined with paclitaxel was found to affect the progress of TNBC and improve its prognosis. Accordingly, treatment with atezozumab has been approved by the Food and Drug Administration (FDA) for PD-L1-positive advanced or metastatic TNBC (51). Other immunotherapies for breast cancer, including cytotoxic T-lymphocyte-associated antigen 4 or CTLA-4, tumor-infiltrating lymphocytes, and tumor vaccines, have also achieved relatively good results in the research setting and may pave the way for more breast cancer treatments that can be applied in the clinical setting (52). The combination of cell pyroptosis induction and immunotherapy may synergistically increase anticancer activity (53). Accordingly, it has been indicated that cell-pyroptosis-related gene signatures correlate with almost all steps of the cancer immune cycle (54). The results of immune infiltration analysis in the present study showed that the complex prognostic values correlated positively with the infiltration of naive B cells, CD8+ T cells, and mast cells, while they correlated negatively with M0 macrophages and dendritic cells. Thus, cell pyroptosis pathways may have an impact on the immune-oncology landscape. Finally, the data obtained from the GDSC database indicated that patients with increased expression of the ZMYND10, PCP2, TNN, and FREM1 genes may not respond well to treatment with XAV939 and dastatinib; however, they may respond to treatment with AS605240, FK886, etc.

Some limitations of this study need to be mentioned. As the data were obtained from public databases, they need to be validated with laboratory/real clinical data. Yu et al. have identified different pyroptosis genes from us in humans through the TCGA and GEO databases (55), but the good thing is that those pyroptosis genes found by both of us can predict the prognosis of breast cancer. Therefore, in the next study, we will analyze the reported data and verify the relationship between pyroptosis genes and breast cancer through *in vivo* and *in vitro* experiments. However, the immune microenvironment has a very important impact on tumors, so we will also focus on the regulation of pyroptosis genes in the immune microenvironment and the effects on related immune cells. In conclusion, our results suggest that induction of cell pyroptosis may be a novel strategy for breast cancer immunotherapy and has broad clinical applicability. The findings may provide an important basis for future studies in terms of developing efficient treatment strategies for breast cancer.

## Data availability statement

Publicly available datasets were analyzed in this study. The data can be found here: GSE96058 dataset at https://www.ncbi.nlm.nih.gov/geo/, TCGA dataset at https://xenabrowser.net/datapages/, METABRIC dataset at http://www.cbioportal.org/, and GDSC drug data at https://www.cancerrxgene.org.

## Ethics statement

Ethical review and approval were not required for this study, in accordance with the local legislation and institutional requirements. Written informed consent for participation was also not required in accordance with the national legislation and the institutional requirements. Therefore, written informed consent was not obtained from the individual(s) for the publication of any potentially identifiable images or data included in this article.

## Author contributions

CW, LZ, LR, NS, YZ, and XQ conceived and designed the analysis and implemented the experimental studies. CW performed statistical analysis, interpreted the results, graphed the data, and wrote the paper. GZ, AW, SX , HT, ZP, TZ, and PG modified the draft. NS, YZ, and XQ approved the draft of the paper. All authors contributed to the article and approved the submitted version.

## Funding

This study was funded by grants from Army Medical University (No. XZ-2019-505-042), Chongqing Basic and Frontier Research Exploration Project (No. cstc2018jcyjA0137), and Chongqing Talent Program Project (No. CQYC20210303406).

## Conflict of interest

The authors declare that the research was conducted in the absence of any commercial or financial relationships that could be construed as a potential conflict of interest.

## Publisher’s note

All claims expressed in this article are solely those of the authors and do not necessarily represent those of their affiliated organizations, or those of the publisher, the editors and the reviewers. Any product that may be evaluated in this article, or claim that may be made by its manufacturer, is not guaranteed or endorsed by the publisher.

## Supplementary material

Supplementary Figure S1 The principal component analysis(PCA) results for **(A)** tumor/normal and **(B)** survival time.

Supplementary Figure S2 Analysis of the association between survival and expression based on the complex prognostic values. **(A)** Overall sample, **(B)** LumB status, **(C)** Basal status, **(D)** LumA status, **(E)** Normal, **(F)** Her2 status.

Supplementary Table S1 Pyroptosis-related genes from the PMID:338828074, MsigDB, and GeneCards databases.

Supplementary Table S2 List of screened 57 pyroptosis genes.

Supplementary Table S3 RNA expression profile data set of 4996 pyroptosis-related genes for breast cancer.

Supplementary Table S4 Differentially expressed genes between breast cancer and normal tissue samples.

Supplementary Table S5 Differentially expressed genes between long-term survival and short-term survival.

Supplementary Table S6 GO analysis of the key functional genes.

Supplementary Table S7 KEGG analysis of the key functional genes.

Supplementary Table S8 GSEA analysis of the key functional genes.

Supplementary Table S9 Docking scores. According to the docking score, 5645 small-molecule compounds were sorted according to their binding abilities.

## References

[1] Ferlay J Colombet M Soerjomataram I Parkin DM Piñeros M Znaor A. Cancer statistics for the year 2020: An overview. Int J Cancer (2021) 149(4):778–89. doi: 10.1002/ijc.33588 33818764

[2] Sung H Ferlay J Siegel RL Laversanne M Soerjomataram I Jemal A. Global cancer statistics 2020: GLOBOCAN estimates of incidence and mortality worldwide for 36 cancers in 185 countries. CA: Cancer J For Clin (2021) 71(3):209–49. doi: 10.3322/caac.21660 33538338

[3] Sopik V Sun P Narod SA. Predictors of time to death after distant recurrence in breast cancer patients. Breast Cancer Res Treat (2019) 173(2):465–74. doi: 10.1007/s10549-018-5002-9 30328050

[4] Heilig R Dick MS Sborgi L Meunier E Hiller S Broz P. The gasdermin-d pore acts as a conduit for IL-1β secretion in mice. Eur J Immunol (2018) 48(4):584–92. doi: 10.1002/eji.201747404 29274245

[5] Zychlinsky A Prevost MC Sansonetti PJ. Shigella flexneri induces apoptosis in infected macrophages. Nature (1992) 358(6382):167–9. doi: 10.1038/358167a0 1614548

[6] Cookson BT Brennan MA. Pro-inflammatory programmed cell death. Trends In Microbiol (2001) 9(3):113–4. doi: 10.1016/S0966-842X(00)01936-3 11303500

[7] Ding J Wang K Liu W She Y Sun Q Shi J. Pore-forming activity and structural autoinhibition of the gasdermin family. Nature (2016) 535(7610):111–6. doi: 10.1038/nature18590 27281216

[8] Li L Li Y Bai Y. Role of GSDMB in pyroptosis and cancer. Cancer Manage Res (2020) 12:3033–43. doi: 10.2147/CMAR.S246948 PMC7201009 32431546

[9] Sarhan J Liu BC Muendlein HI Li P Nilson R Tang AY. Caspase-8 induces cleavage of gasdermin d to elicit pyroptosis during infection. Proc Natl Acad Sci United States America (2018) 115(46):E10888–97. doi: 10.1073/pnas.1809548115 PMC6243247 30381458

[10] Xiao J Wang C Yao JC Alippe Y Xu C Kress D. Gasdermin d mediates the pathogenesis of neonatal-onset multisystem inflammatory disease in mice. PloS Biol (2018) 16(11):e3000047. doi: 10.1371/journal.pbio.3000047 30388107 PMC6235378

[11] Jiang M Wu Y Qi L Li L Song D Gan J. Dihydroartemisinin mediating PKM2-caspase-8/3-GSDME axis for pyroptosis in esophageal squamous cell carcinoma. Chemico-biological Interact (2021) 350:109704. doi: 10.1016/j.cbi.2021.109704 34655567

[12] Rioja-Blanco E Arroyo-Solera I Álamo P Casanova I Gallardo A Unzueta U. CXCR4-targeted nanotoxins induce GSDME-dependent pyroptosis in head and neck squamous cell carcinoma. J Exp Clin Cancer Res CR (2022) 41(1):49. doi: 10.1186/s13046-022-02267-8 PMC8815235 35120582

[13] Hage C Hoves S Strauss L Bissinger S Prinz Y Pöschinger T. Sorafenib induces pyroptosis in macrophages and triggers natural killer cell-mediated cytotoxicity against hepatocellular carcinoma. Hepatol (Baltimore Md.) (2019) 70(4):1280–97. doi: 10.1002/hep.30666 31002440

[14] Ma X Hao J Wu J Li Y Cai X Zheng Y. Prussian Blue nanozyme as a pyroptosis inhibitor alleviates neurodegeneration. Advanced Materials (Deerfield Beach Fla.) (2022) 34(15):e2106723. doi: 10.1002/adma.202106723 35143076

[15] Liu X Xia S Zhang Z Wu H Lieberman J. Channelling inflammation: gasdermins in physiology and disease. Nat Rev Drug Discov (2021) 20(5):384–405. doi: 10.1038/s41573-021-00154-z PMC7944254 33692549

[16] Du T Gao J Li P Wang Y Qi Q Liu X. Pyroptosis, metabolism, and tumor immune microenvironment. Clin Trans Med (2021) 11(8):e492. doi: 10.1002/ctm2.492 PMC8329701 34459122

[17] Peng Z Wang P Song W Yao Q Li Y Liu L. GSDME enhances cisplatin sensitivity to regress non-small cell lung carcinoma by mediating pyroptosis to trigger antitumor immunocyte infiltration. Signal Transduct Target Ther (2020) 5(1):159. doi: 10.1038/s41392-020-00274-9 PMC7445264 32839451

[18] Hou J Zhao R Xia W Chang CW You Y Hsu JM. PD-L1-mediated gasdermin c expression switches apoptosis to pyroptosis in cancer cells and facilitates tumour necrosis. Nat Cell Biol (2020) 22(10):1264–1275. doi: 10.1038/s41556-020-0575-z PMC7653546 32929201

[19] Wu D Wei C Li Y Yang X Zhou S. Pyroptosis, a new breakthrough in cancer treatment. Front Oncol (2021) 11:698811. doi: 10.3389/fonc.2021.698811 34381721 PMC8350724

[20] Meng J Huang X Qiu Y Zheng X Huang J Wen Z. Pyroptosis-related gene mediated modification patterns and immune cell infiltration landscapes in cutaneous melanoma to aid immunotherapy. Aging (2021) 13(21):24379–401. doi: 10.18632/aging.203687 PMC8610130 34753832

[21] Wang H Rong X Zhao G Zhou Y Xiao Y Ma D. The microbial metabolite trimethylamine n-oxide promotes antitumor immunity in triple-negative breast cancer. Cell Metab (2022) 34(4):581–594.e8. doi: 10.1016/j.cmet.2022.02.010 35278352

[22] Xia C Dong X Li H Cao M Sun D He S. Cancer statistics in China and United States, 2022: profiles, trends, and determinants. Chin Med J (2022) 135(5):584–90. doi: 10.1097/CM9.0000000000002108 PMC8920425 35143424

[23] Soerjomataram I Bray F. Planning for tomorrow: global cancer incidence and the role of prevention 2020-2070. Nat Rev Clin Oncol (2021) 18(10):663–72. doi: 10.1038/s41571-021-00514-z 34079102

[24] Loveless R Bloomquist R Teng Y. Pyroptosis at the forefront of anticancer immunity. J Exp Clin Cancer Res CR (2021) 40(1):264. doi: 10.1186/s13046-021-02065-8 PMC8383365 34429144

[25] Ping L Zhang K Ou X Qiu X Xiao X. A novel pyroptosis-associated long non-coding RNA signature predicts prognosis and tumor immune microenvironment of patients with breast cancer. Front In Cell Dev Biol (2021) 9:727183. doi: 10.3389/fcell.2021.727183 34616734 PMC8488148

[26] Zhang Z Zhang H Li D Zhou X Qin Q Zhang Q. Caspase-3-mediated GSDME induced pyroptosis in breast cancer cells through the ROS/JNK signalling pathway. J Cell Mol Med (2021) 25(17):8159–68. doi: 10.1111/jcmm.16574 PMC8419174 34369076

[27] Molina-Crespo Á Cadete A Sarrio D Gámez-Chiachio M Martinez L Chao K. Intracellular delivery of an antibody targeting gasdermin-b reduces HER2 breast cancer aggressiveness. Clin Cancer Res (2019) 25(15):4846–58. doi: 10.1158/1078-0432.CCR-18-2381. 31064780

[28] Tan Y Sun R Liu L Yang D Xiang Q Li L. Tumor suppressor DRD2 facilitates M1 macrophages and restricts NF-κB signaling to trigger pyroptosis in breast cancer. Theranostics (2021) 11(11):5214–31. doi: 10.7150/thno.58322 PMC8039962 33859743

[29] van der Zanden SY Luimstra JJ Neefjes J Borst J Ovaa H. Opportunities for small molecules in cancer immunotherapy. Trends Immunol (2020) 41(6):493–511. doi: 10.1016/j.it.2020.04.004 32381382

[30] Mancini RJ Stutts L Ryu KA Tom JK Esser-Kahn AP. Directing the immune system with chemical compounds. ACS Chem Biol (2014) 9(5):1075–85. doi: 10.1021/cb500079s PMC5674983 24690004

[31] Khera N Rajput S. Therapeutic potential of small molecule inhibitors. J Cell Biochem (2017) 118(5):959–961. doi: 10.1002/jcb.25782 27813176

[32] Gu J Li K Li M Wu X Zhang L Ding Q. A role for p21-activated kinase 7 in the development of gastric cancer. FEBS J (2013) 280(1):46–55. doi: 10.1111/febs.12048 23106939

[33] He S Liu M Zhang W Xu N Zhu H. Over expression of p21-activated kinase 7 associates with lymph node metastasis in esophageal squamous cell cancers. Cancer Biomarkers Section A Dis Markers (2016) 16(2):203–9. doi: 10.3233/CBM-150557 PMC13016460 26682509

[34] Li K Xu X He Y Tian Y Pan W Xu L. P21-activated kinase 7 (PAK7) interacts with and activates wnt/β-catenin signaling pathway in breast cancer. J Cancer (2018) 9(10):1821–35. doi: 10.7150/jca.24934 PMC5968771 29805709

[35] Song X Xie Y Liu Y Shao M Yang W. MicroRNA-492 overexpression exerts suppressive effects on the progression of osteosarcoma by targeting PAK7. Int J Mol Med (2017) 40(3):891–7. doi: 10.3892/ijmm.2017.3046 28677719

[36] Zeng H Tong F Bin Y Peng L Gao X Xia X. The predictive value of PAK7 mutation for immune checkpoint inhibitors therapy in non-small cell cancer. Front In Immunol (2022) 13:834142. doi: 10.3389/fimmu.2022.834142 35242138 PMC8886445

[37] Fang H Liu Y He Y Jiang Y Wei Y Liu H. Extracellular vesicle−delivered miR−505−5p, as a diagnostic biomarker of early lung adenocarcinoma, inhibits cell apoptosis by targeting TP53AIP1. Int J Oncol (2019) 54(5):1821–32. doi: 10.3892/ijo.2019.4738. 30864684

[38] Wang Y Kanneganti T-D. From pyroptosis, apoptosis and necroptosis to PANoptosis: A mechanistic compendium of programmed cell death pathways. Comput Struct Biotechnol J (2021) 19:4641–57. doi: 10.1016/j.csbj.2021.07.038 PMC8405902 34504660

[39] Wang Y Dan L Li Q Li L Zhong L Shao B. ZMYND10, an epigenetically regulated tumor suppressor, exerts tumor-suppressive functions *via* miR145-5p/NEDD9 axis in breast cancer. Clin Epigenet (2019) 11(1):184. doi: 10.1186/s13148-019-0785-z PMC6894283 31801619

[40] Dong SM Byun HJ Kim BR Lee SH Trink B Rho SB. Tumor suppressor BLU enhances pro-apoptotic activity of sMEK1 through physical interaction. Cell Signalling (2012) 24(6):1208–14. doi: 10.1016/j.cellsig.2012.02.002 22349239

[41] Li HN Li XR Lv ZT Cai MM Wang G Yang ZF. Elevated expression of FREM1 in breast cancer indicates favorable prognosis and high-level immune infiltration status. Cancer Med (2020) 9(24):9554–70. doi: 10.1002/cam4.3543 PMC7774739 33058542

[42] Xu XY Guo WJ Pan SH Zhang Y Gao FL Wang JT. TILRR (FREM1 isoform 2) is a prognostic biomarker correlated with immune infiltration in breast cancer. Aging (2020) 12(19):19335–51. doi: 10.18632/aging.103798 PMC7732299 33031059

[43] Zhang X Pino GM Shephard F Kiss-Toth E Qwarnstrom EE. Distinct control of MyD88 adapter-dependent and akt kinase-regulated responses by the interleukin (IL)-1RI co-receptor, TILRR. J Biol Chem (2012) 287(15):12348–52. doi: 10.1074/jbc.C111.321711 PMC3320984 22262840

[44] McGregor L Makela V Darling SM Vrontou S Chalepakis G Roberts C. Fraser Syndrome and mouse blebbed phenotype caused by mutations in FRAS1/Fras1 encoding a putative extracellular matrix protein. Nat Genet (2003) 34(2):203–8. doi: 10.1038/ng1142 12766769

[45] Kang S Maeng H Kim BG Qing GM Choi YP Kim HY. *In situ* identification and localization of IGHA2 in the breast tumor microenvironment by mass spectrometry. J Proteome Res (2012) 11(9):4567–74. doi: 10.1021/pr3003672 22894699

[46] Liu HR Jiang GZ Xin D Yang YL Fan QX Meng XR. [Establishment and validation of prognostic risk score model for esophageal squamous cell carcinoma based on immune related genes]. Zhonghua Zhong Liu Za Zhi [Chinese J Oncology] (2021) 43(6):666–73. doi: 10.3760/cma.j.cn112152-20200917-00831 34289558

[47] Ye T Haoyuan Z Bei Z Kangyong X. Exploration of biomarkers in osteoarthritis based on bioinformatics. Medicine (2021) 100(31):e26730. doi: 10.1097/MD.0000000000026730 34397812 PMC8341221

[48] Bender S Javaugue V Saintamand A Ayala MV Alizadeh M Filloux M. Immunoglobulin variable domain high-throughput sequencing reveals specific novel mutational patterns in POEMS syndrome. Blood (2020) 135(20):1750–8. doi: 10.1182/blood.2019004197 PMC8209552 32243509

[49] Ramirez-Perez S Oregon-Romero E Reyes-Perez IV Bhattaram P. Targeting MyD88 downregulates inflammatory mediators and pathogenic processes in PBMC from DMARDs-naïve rheumatoid arthritis patients. Front Pharmacol (2021) 12:800220. doi: 10.3389/fphar.2021.800220 35002734 PMC8735861

[50] Jang B-S Han W Kim IA. Tumor mutation burden, immune checkpoint crosstalk and radiosensitivity in single-cell RNA sequencing data of breast cancer. Radiotherapy Oncol J Eur Soc For Ther Radiol Oncol (2020) 142:202–9. doi: 10.1016/j.radonc.2019.11.003 31767471

[51] Blackley EF Loi S. Targeting immune pathways in breast cancer: review of the prognostic utility of TILs in early stage triple negative breast cancer (TNBC). Breast (2019) 48(Suppl 1):S44–8. doi: 10.1016/S0960-9776(19)31122-1 31839159

[52] Zhang D Xu X Ye Q. Metabolism and immunity in breast cancer. Front Med (2021) 15(2):178–207. doi: 10.1007/s11684-020-0793-6 33074528

[53] Wang Q Wang Y Ding J Wang C Zhou X Gao W. A bioorthogonal system reveals antitumour immune function of pyroptosis. Nature (2020) 579(7799):421–6. doi: 10.1038/s41586-020-2079-1 32188939

[54] Cao J Yan Q. Cancer epigenetics, tumor immunity, and immunotherapy. Trends Cancer (2020) 6(7):580–92. doi: 10.1016/j.trecan.2020.02.003 PMC7330177 32610068

[55] Yu H Fu Y Tang Z Jiang L Qu C Li H. A novel pyroptosis-related signature predicts prognosis and response to treatment in breast carcinoma. Aging (Albany NY) (2022) 14(2):989–1013. doi: 10.18632/aging.203855 PMC8833126 35085103

