# Supplementary figures and images for: A novel pyroptosis-related indicator of immune infiltration features and prognosis in breast cancer
Source: Front Oncol. 2022 Sep 7;12:961500. doi: 10.3389/fonc.2022.961500 (PMC9491236; doi:10.3389/fonc.2022.961500)

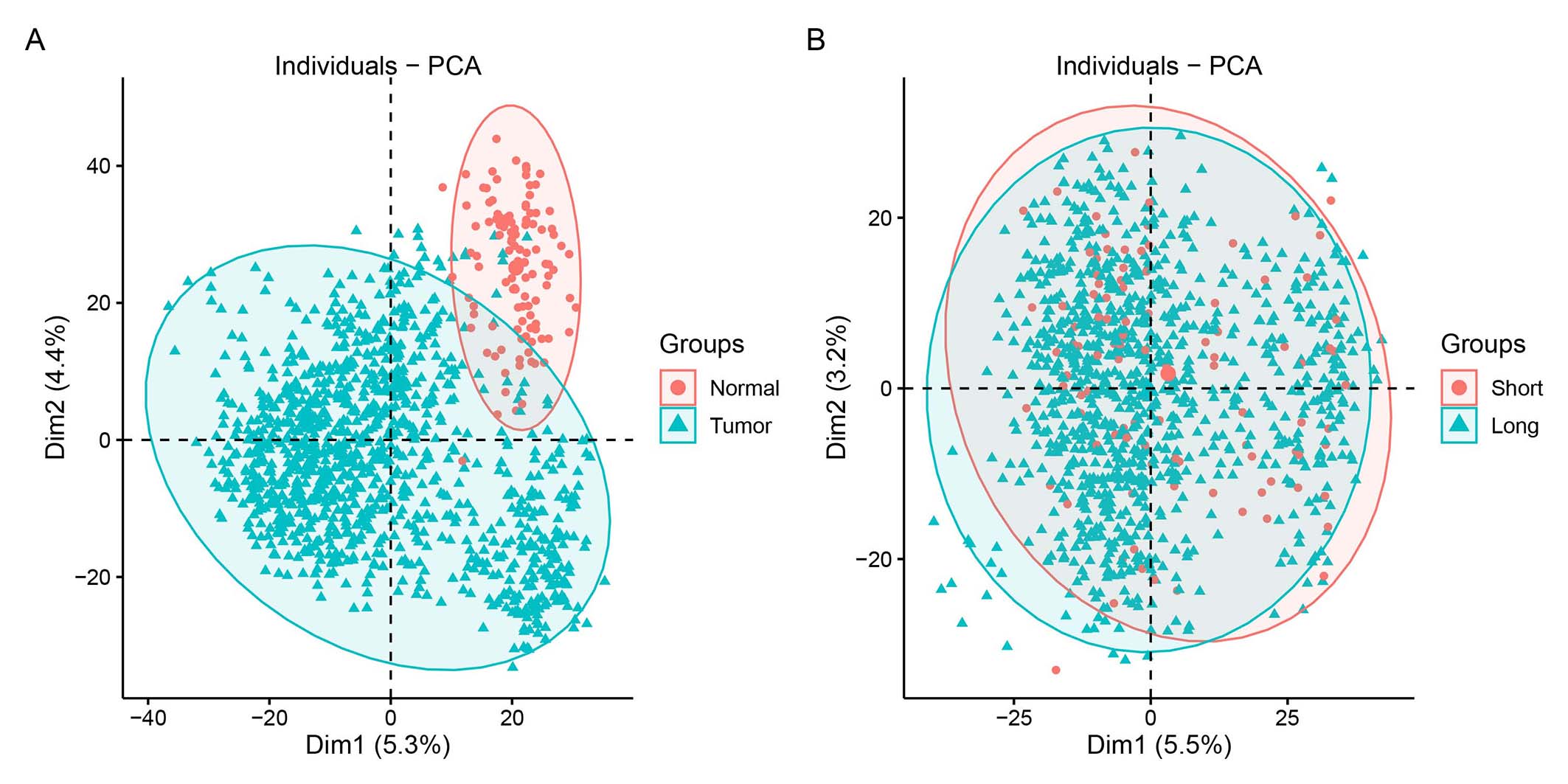

Supplement: Supplementary Figure S1 — The principal component analysis(PCA) results for (A) tumor/normal and (B) survival time. [file Image_1.jpeg]

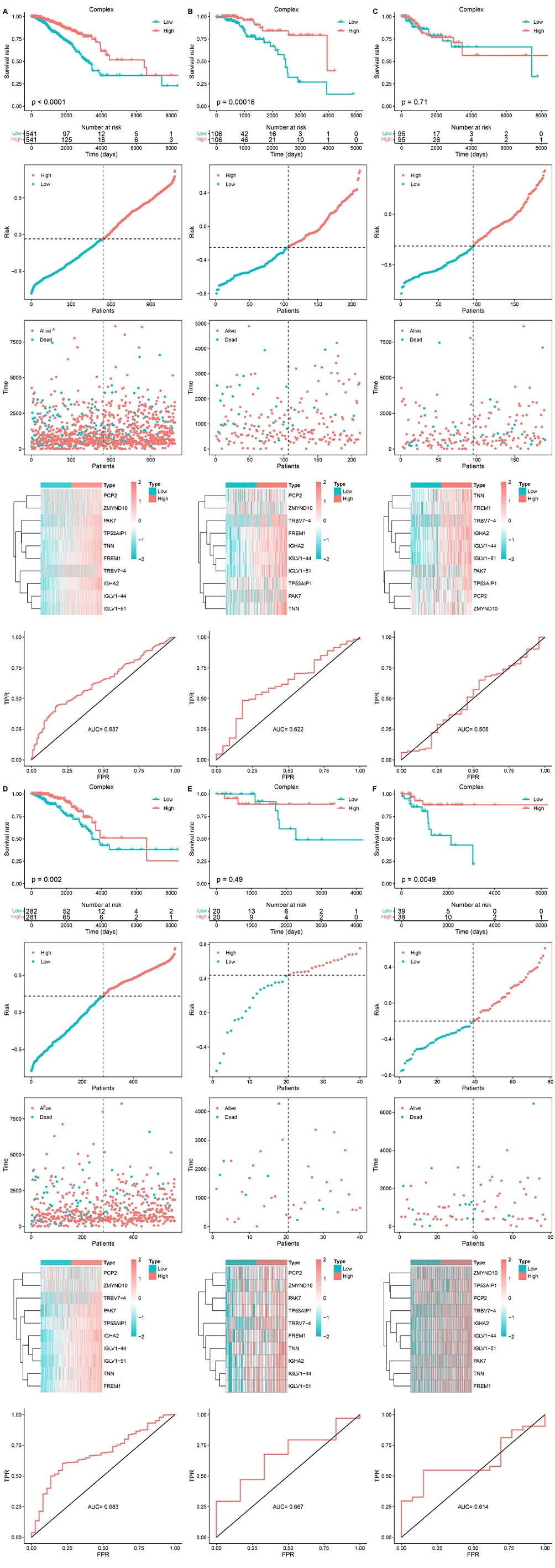

Supplement: Supplementary Figure S2 — Analysis of the association between survival and expression based on the complex prognostic values. (A) Overall sample, (B) LumB status, (C) Basal status, (D) LumA status, (E) Normal, (F) Her2 status. [file Image_2.jpeg]
